# Measles Virus Fusion Protein: Structure, Function and Inhibition

**DOI:** 10.3390/v8040112

**Published:** 2016-04-21

**Authors:** Philippe Plattet, Lisa Alves, Michael Herren, Hector C. Aguilar

**Affiliations:** 1 Division of Experimental and Clinical Research, , Vetsuisse Faculty, University of Bern, Bremgartenstrasse 109a, Bern 3001, Switzerland; michael.herren@vetsuisse.unibe.ch; 2 Clinical Neurology, Department of Veterinary Medicine, University of Cambridge, CB3 0ES, Cambridge, UK; calves.lisa@gmail.com; 3 Graduate School for Cellular and Biomedical Sciences, University of Bern, Bern 3012, Switzerland; 4 Paul G. Allen School for Global Animal Health, Washington State University, Pullman, WA 99164, USA; haguilar@vetmed.wsu.edu; 5 Department of Veterinary Microbiology and Pathology, Washington State University, Pullman, WA 99164, USA

**Keywords:** measles virus, cell entry, fusion protein, structural changes, inhibitors and mechanisms of adaptation, neuroinvasion, membrane fusion

## Abstract

Measles virus (MeV), a highly contagious member of the *Paramyxoviridae* family, causes measles in humans. The *Paramyxoviridae* family of negative single-stranded enveloped viruses includes several important human and animal pathogens, with MeV causing approximately 120,000 deaths annually. MeV and canine distemper virus (CDV)-mediated diseases can be prevented by vaccination. However, sub-optimal vaccine delivery continues to foster MeV outbreaks. Post-exposure prophylaxis with antivirals has been proposed as a novel strategy to complement vaccination programs by filling herd immunity gaps. Recent research has shown that membrane fusion induced by the morbillivirus glycoproteins is the first critical step for viral entry and infection, and determines cell pathology and disease outcome. Our molecular understanding of morbillivirus-associated membrane fusion has greatly progressed towards the feasibility to control this process by treating the fusion glycoprotein with inhibitory molecules. Current approaches to develop anti-membrane fusion drugs and our knowledge on drug resistance mechanisms strongly suggest that combined therapies will be a prerequisite. Thus, discovery of additional anti-fusion and/or anti-attachment protein small-molecule compounds may eventually translate into realistic therapeutic options.

## 1. Introduction

Measles virus (MeV) induces measles in humans. MeV is a highly contagious enveloped virus carrying a negative single-stranded RNA genome and belongs to the *Morbillivirus* genus within the *Paramyxoviridae* family. The *Paramyxoviridae* family is divided into two subfamilies: *Paramyxovirinae* and *Pneumovirinae*. The *Paramyxovirinae* subfamily is further divided into seven genera: *Avulavirus*, *Morbillivirus*, *Respirovirus*, *Rubulavirus*, *Henipavirus*, *Aquaparamyxovirus* and *Ferlavirus*, whereas *Pneumovirinae* is composed of two genera: *Pneumovirus* and *Metapneumovirus* [1].

The *Paramyxoviridae* family includes several important pathogens responsible for high morbidity and variable mortality among humans and animals. In humans, MeV, mumps virus (MuV), human parainfluenza virus (hPIV), respiratory syncytial virus (RSV), and human metapneumovirus (hMPV) cause prevalent diseases, with MeV being responsible for approximately 120,000 deaths annually [2,3]. Furthermore, henipaviruses (Nipah virus (NiV) and Hendra virus (HeV)) can infect both animals and humans and are associated with high mortality rates, hence representing a zoonotic threat [4,5,6,7].

In veterinary medicine, several members of the *Morbillivirus* genus are major pathogens. Canine distemper virus (CDV) causes a widespread disease in domestic carnivores and is responsible for fatal outbreaks in wildlife [8,9,10,11,12,13]. Whilst rinderpest virus (RPV) has been eradicated [14], peste-des-petits-ruminants virus (PPRV) still causes important losses in African and Asian goats and sheep [15], and in recent years, the aquatic mammal morbilliviruses (Phocine distemper virus (PDV) and cetacean morbilliviruses (CeMV)) were responsible for dramatic epidemics in wild pinnipeds and cetaceans [16,17]. Other paramyxoviruses outside of the *Morbillivirus* genus, such as Newcastle disease virus (NDV), bovine respiratory syncytial virus (bRSV), and avian metapneumovirus (AMPV) continue to have a serious impact on animal health and world economics [1].

Both MeV and CDV-mediated diseases can be prevented by vaccination and global MeV eradication has been considered feasible if 95% herd immunity could be achieved [18]. Although targeted for eradication, in 2014 MeV was still associated with more than 120,000 deaths worldwide [19,20,21]. However, sub-optimal vaccine delivery in developing countries and vaccination refusal induced by unfounded anxiety concerning the vaccine’s safety in western countries continue to foster MeV outbreaks. During the last years, the number of MeV outbreaks in USA has been steadily increasing, and the recent outbreak in Disneyland showcased the importance of sustaining vaccination campaigns.

Recently, in order to achieve the World Health Organization (WHO)-targeted global MeV eradication, post-exposure prophylaxis with antivirals has been proposed as a novel strategy aiming at complementing vaccination programs by filling herd immunity gaps [3]. Indeed, immediate treatment with antiviral compounds of people exposed to confirmed patients with measles may contribute to prevent further viral transmission and, thus, prevent an epidemic. This is an attractive strategy especially because MeV-infected patients present a two-week asymptomatic period before becoming contagious, thereby offering an excellent window of opportunity for successful prophylactic interventions. Additionally, and of major importance, blocking MeV outbreaks would likely be beneficial to combat other infectious diseases. Indeed, Mina and colleagues recently suggested that long-term MeV-induced immunomodulation enhances the risk of death due to non-measles infections [22].

Although two inhibitors were recently demonstrated to be efficient in animal models of morbillivirus-induced disease [23,24,25], Food and Drug Administration (FDA)-approved anti-MeV drugs are currently not yet available on the market, thus underlining the need for the development of additional therapeutic drugs. Moreover, due to a considerable risk of emergence of drug-resistant viruses, the development of combined therapies with antiviral compounds is indicated.

Paramyxoviruses have two viral glycoproteins, the attachment glycoprotein (HN, H or G) and the fusion glycoprotein (F). Although F proteins from members of the *Pneumovirinae* subfamily share many similarities with those encoded by members of the *Paramyxovirinae* subfamily, their respective attachment glycoproteins (Gs) are structurally and likely functionally more distinct [1,26]. For this reason, this review will mainly focus on and compare the MeV F protein with other paramyxovirinae F proteins.

## 2. The Diseases

The pathogenesis of MeV and CDV is very similar. Both viruses enter their hosts through the respiratory tract and target immune cells residing within the airways [27,28,29,30,31]. After the ensuing massive amplification in lymphoid organs, which is associated with profound immunosuppression potentially fostering secondary bacterial infections, both viruses disseminate via the blood stream to multiple organs leading to gastrointestinal, dermatological and respiratory signs [32].

Viremia may also lead to central nervous system (CNS) invasion whereby MeV and CDV can induce fatal brain disorders [33,34]. Of note, it has been reported that both viruses, upon CNS invasion, likely spread from cell-to-cell in a non-cytolytic manner (without the formation of syncytia); a phenotype that presumably contributes to the induction of CNS lesions. Indeed, non-cytolytic cell-to-cell viral transmission represents a mode of lateral dissemination totally different from the one induced by the same viruses in other tissues where large multinucleated cells are formed (see Section 3). Although encephalitis of the grey matter is also observed, brain infections with CDV in dogs usually lead to chronic progressive or relapsing multifocal demyelination, which resembles multiple sclerosis in humans. MeV can induce primary measles encephalitis, acute post-infectious measles encephalomyelitis and MeV inclusion body encephalitis (MIBE). It is remarkable that both viruses can induce a rare diffuse progressive *pan*-encephalitis, which is known as subacute sclerosing panencephalitis (SSPE) in humans [35] and old dog encephalitis (ODE) in canines [34]. However, while brain complications represent a hallmark of CDV infections, they remain very limited in the case of MeV infections.

## 3. Cell Entry: The Viral Membrane Fusion Machinery

Viruses are strictly cell-dependent organisms for replication and, therefore, entering a host cell constitutes a pivotal event in the course of an infection. Armed with the fusion machinery, morbilliviruses can successfully bypass the first line of defense of the host cell: the plasma membrane. The morbillivirus fusion machinery is composed of two viral surface proteins forming hetero-oligomeric complexes. Complexes are composed of tetrameric attachment (H) and trimeric fusion (F) glycoproteins. It is believed that upon interaction of H with a specific host cell receptor, a sequential cascade of conformational changes is triggered (first in H and then in F). Ultimately, these structural rearrangements lead to viral-cell membrane merging, fusion pore formation and enlargement, and injection of the ribonucleocapsid complex into the host cell cytosol [1]. Importantly, membrane fusion is not only required for virus-to-cell entry but also for lateral cell-to-cell spread, which eventually results in multinucleated cell formation (also referred to as syncytia), a hallmark of morbilliviral and many other paramyxoviral infections.

## 4. Host Cellular Receptors and Pathogenesis

For viral-cell entry and, presumably, for regular cell-to-cell viral spread (with syncytia formation), an interaction with the host cellular receptor is mandatory; a task that is specifically carried out by the viral attachment H glycoprotein of morbilliviruses and the G or HN glycoproteins of other paramyxoviral genera. While paramyxoviruses carrying an HN attachment glycoprotein (such as hPIV5, hPIV3 or NDV) detect sialic acid-containing receptors, morbilliviruses and henipaviruses recognize protein receptors. This and other features (discussed below) suggest relative similarities in the membrane fusion mechanisms between the morbilliviruses and the henipaviruses as opposed to other paramyxoviral genera. The attachment protein of henipaviruses (G) binds to the ephrin receptors ephrin-B2 and ephrin-B3 [36,37,38], which are largely expressed on vascular endothelial cells and neurons, largely correlating with viral tropism and pathology [39]. For morbilliviruses, two receptors have been reported, the CD150 or signaling lymphocyte activation molecule (SLAM) [40,41] and the nectin-4 (N4) or poliovirus receptor-like protein 4 (PVRL4) [42,43,44,45]. The localization of SLAM and N4 on specific cells nicely correlates with the morbillivirus-induced pathology. Expression of SLAM on activated immune cells [46] partly explains the observed severe immunosuppression in morbillivirus infections. On the other hand, N4, which is expressed on the basolateral side of epithelial cells, not only explains the *in vivo* epithelio-tropism of MeV and other morbilliviruses, but also why these viruses cannot directly access the airway epithelia by the respiratory route [43]. Rather, morbilliviruses initially infect SLAM-positive macrophages and/or dendritic cells residing within the airways [31,47], which subsequently enable viral amplification throughout the lymphatic tissues. The ensuing viremia then presumably allows targeting the N4-positive basolateral surface of epithelial cells of many organs, including the airways [48]. Release of infectious particles from the luminal surface of epithelial cells in the respiratory tract leads to spread of the infection in the environment through coughing and sneezing. Interestingly, CD46, a membrane bound protein expressed in all nucleated cells, was the first reported functional receptor for MeV [49]. However, it was later demonstrated that CD46 was only used by MeV vaccine strains; a capacity that very likely emerged from multiple passages of wild-type viruses in tissue cultures [50]. The usage of human CD46 or a specific orthologue molecule as an entry receptor for other morbilliviruses remains unclear and deserves further investigation.

In addition to the above described lympho- and epithelio-tropism, MeV and CDV are also well-known to be neurotropic [35,51], although neurological complications are far more frequent in distemper than in measles [34]. Remarkably, in the CNS, both viruses were reported to spread from cell-to-cell seemingly without formation of free viral particles and presumably by microfusions between infected and target cells, thus spotlighting a unique mode of morbillivirus dissemination [52,53,54,55,56]. Although it has been proposed that an unknown cell surface receptor in neurons might be targeted by the MeV H protein for entry, subsequent non-cytolytic neuron-to-neuron viral transmission was postulated to be controlled by the F protein alone [56]. F may directly contact a specific membrane-bound molecule, which would be sufficient to trigger its refolding and the ensuing membrane fusion process [57]. The latter postulated microfusion events (also called intercellular membrane pores) have been recently proposed to be also induced by MeV in well-differentiated human airway epithelial cells enabling rapid viral spread in the upper respiratory epithelium [58].

In sharp contrast, CDV-mediated non-cytolytic lateral spread in primary cultures of canine astrocytes was reported to be strictly dependent on the H protein [53]. Moreover, growing evidence strongly suggests that astrocyte-to-astrocyte nucleocapsid transmission relies on viral functional fusion machineries that are activated by proper H/receptor contacts [59]. Because both SLAM and N4 were recently excluded as candidate entry molecules, the putative CDV receptor in astrocytes was arbitrarily referred to as “GliaR” awaiting precise characterization of this surface molecule [59].

While there are hardly any doubts that the membrane fusion machinery plays an instrumental role in the initiation of a broad range of morbillivirus-induced pathologies, increasing evidence highlights a central role of the F protein in fine-tuning the fusion machinery. We propose that variations in degree of membrane fusion triggering (ranging from microfusion pores to extensive syncytia formation) may lead to essential differences in ribonucleocapsid transmission dynamics in infected tissues. The latter may translate into specific primary virally induced pathological changes as well as immunopathological complications in the frame of the antiviral immune response: two major factors that certainly shape the clinical course and outcome of an infection.

## 5. The Fusion Protein F

### 5.1. General Information

**Figure 1:**
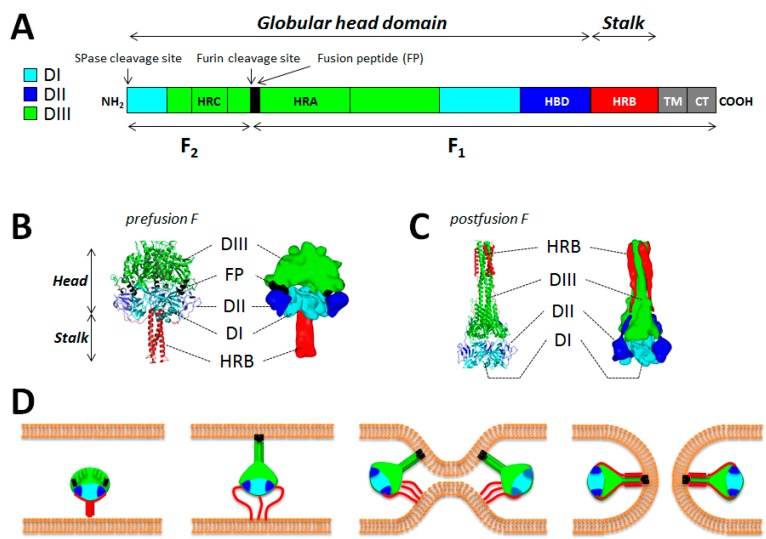
**Structure/function of the measles virus (MeV) fusion protein.** (**A**) The important functional domains of F are color-coded. The F_1_ and F_2_ subunits are shown. While the DI (light blue), DII (dark blue) and DIII (green) domains define the globular head domain, the HRB region defines the stalk. TM: transmembrane domain; CT: cytoplasmic tail; HRA-C: heptad repeat region A–C; FP: fusion peptide; HBD: H-binding domain; SPase: signal peptidase. (**B** and **C**) Structural differences between the pre and postfusion states of F. Images were obtained from homology models performed with the canine distemper virus (CDV) F sequence (strain A75/17) and based on the atomic coordinates of the human parainfluenza virus type 5 (hPIV5) F prefusion state (PDB 2B9B) and hPIV3 F postfusion state (PDB 1ZTM). DI, DII and DIII are color-coded as indicated in (**A**). Images were generated using either the PyMol (high resolution) or Sculptor (low resolution) software packages (**D**) Model of membrane fusion induced by measles virus F. Once activated by H, prefusion F may disengage from H interaction. Then the HRB region of the stalk may melt, which consequently may enable the 11 segments positioned in the globular head to rearrange into a long three-helix bundle (3HB) coiled-coil. This would in turn propel the fusion peptide at the top of the 3HB and allow its insertion into the target plasma membrane. This prehairpin intermediate then collapses, which brings both membranes into close proximity. Finally, F may reach the highly stable postfusion state, which carries the six-helix bundle (6HB) formed by HRA and HRB segments. This stage is thought to correlate with membrane merging and ensuing fusion pore opening. This model likely requires the concerted action of more than one F molecules. This model has been described first by Yin and colleagues [79,86].

The fusion protein (F) is one of the key components of the morbillivirus fusion machinery. As for all paramyxoviruses, MeV F is a type I integral transmembrane protein with a cytoplasmic tail at the C-terminus. Functional F proteins oligomerize as homo-trimers. Each F monomer is first synthesized as a long inactive precursor (F0) that is processed by furin into two disulfide-linked F1 and F2 subunits during its transport through the *trans-*Golgi network (Figure 1A). Furin is a ubiquitous subtilisin-like cellular endoprotease. Typically, morbillivirus F proteins carry multi-basic residues at the cleavage site [1,60]. F trimerization likely occurs in the endoplasmic reticulum (ER), the identical subcellular location where F/H hetero-oligomerization is thought to occur [61], thereby generating so-called membrane fusion machineries. These hetero-oligomeric complexes are finally exposed at the cell surface of the infected cells by traveling through the cellular secretory pathway. For MeV, Brindley and colleagues recently demonstrated that the strength of H/F interaction is strong in the ER compartment and loosened in the Golgi apparatus, as a consequence of F0 cleavage into the functional F1/F2 trimer [62]. Thus, in addition to F0 processing, which is a prerequisite for proper F functionality, the authors proposed that a loosened H/F avidity of interaction (acquired post-F-cleavage) enables enhanced F’s bioactivity.

Paramyxovirus F proteins also belong to class I viral fusion proteins [63,64]. This protein type contains well-conserved regions such as cytoplasmic and transmembrane (TM) domains, two heptad repeat regions A (HRA) and B (HRB) as well as a new *N*-terminal hydrophobic segment (generated upon F0 cleavage), also known as the fusion peptide (FP) [1,65]. MeV F possesses a third heptad repeat region (HRC) located in the F2 subunit that contributes to the formation of a microdomain around residue 94, demonstrated to play a role in modulating fusogenicity (Figure 1A) [66]. Importantly, class I viral fusion proteins are known to initially fold into a structure that contains a high intrinsic energy, termed prefusion or metastable state. The stability of these prefusion structures is proposed to be temporally maintained due to a high intrinsic energy barrier. Importantly, it is believed that upon lowering of the energy barrier, the prefusion state undergoes spontaneous structural rearrangements until reaching a highly stable final structure, termed postfusion state. The postfusion state of class I viral fusion proteins exhibits a lower inherent level of energy and displays a well-characterized central core six-helix bundle (6HB) domain [65,67,68,69].

It is well recognized that paramyxovirus F’s bioactivity can be modulated by their cytoplasmic tails (CTs). While long CT domains have been reported to reduce the ability of F trimers to mediate cell-cell fusion, F complexes carrying short CTs are generally hyperfusogenic [70,71,72]. For NiV F, alanine substitutions in a specific motif (KKR) in the CT domain led to the hyper- or hypo-modulation of the bioactivity through a proposed “inside-out” mechanism [73]. Furthermore, a single tyrosine residue found in the CT domain of MeV F controls its localization to uropod-like structures in polarized lymphocytes and to basolateral surfaces of polarized epithelial cells. The latter residue thus influences the efficiency of viral lateral spread [74], although differences may exist depending on the viral strain studied [75].

Several works additionally reported that the TM of paramyxovirus F proteins could also modulate membrane fusion activity of F complexes, therefore highlighting a role of the TM segment beyond a simple anchor in the lipid bilayer [76]. In the case of MeV F, substitutions performed within the TM region were proposed to influence bioactivity by altering the availability of active membrane fusion machineries at the cell surface [77].

### 5.2. F Structural Information

No structural information is yet available for any morbillivirus F proteins. However, atomic structures of soluble F protein ectodomains in their pre and/or postfusion conformations were recently solved for several paramyxoviruses [78,79,80,81,82,83,84,85,86]. In the case of hPIV5 F, the recently determined crystal structure of the prefusion state highlighted a “tree”-like fold [85,86]. A short stalk region, mainly composed of the three HRB regions assembling in a three-helix bundle (3HB) structure, was found supporting a large globular head domain. The globular head domain has been divided into the three previously identified subdomains per subunit; DI, DII and DIII [80,86]. While DI and DII are forming the base of the head, DIII is mainly constituted of HRA segments that locate at the top of the head structure. In the prefusion state, HRA is composed of eleven segments (four helices, two ß-strands and five loops). Comparison of the pre (hPIV5 F) and postfusion (hPIV3 F) states showed that DI and DII remained mostly unaltered, whereas the eleven HRA segments of DIII were drastically different in both structures (Figure 1B,C). It is also noteworthy that the FP is found mostly buried in the prefusion structure. Indeed, the DII and DIII domains of two different monomers appear to be “clamping” the FP within the globular head domain [86]. The FP is not visible in the postfusion state, but due to the conformation of the 6HB fold, it is believed that both the FP and TM domains may be engaged in physical interactions.

Additionally, a very recent crystal structure of NiV F was reported to consist of hexamer-of-trimers. This higher oligomeric F structure was also observed via biochemical assays and in F-containing viral-like particles (VLPs) by cryo-electron microscopy. Mutations at the trimer-trimer interfaces of the hexamer modulated the fusogenicity of F in both cell-cell fusion and pseudotyped virus entry assays [82]. This hexamer-of-trimer F arrangement could have impactful repercussions in our understanding of triggering of F by the attachment protein, and in fusion pore formation and expansion during the membrane fusion process.

### 5.3. The F-Refolding Cascade

Upon H-mediated triggering, metastable, prefusion F trimers undergo a series of spontaneous and irreversible conformational changes. The cascade of F structural rearrangements is proposed to follow a sequence of several steps: (i) the stalk domain (formed by three compact HRB domains) is thought to open up; (ii) the eleven segments of the HRA region of the three monomers then refold into an extended coiled-coil conformation, thereby allowing the relocation and insertion of the terminal FP into the target membrane, generating the so called “prehairpin” intermediate (PHI) and (iii) the three HRB peptides swing around the base of the globular head to dock onto the coiled-coil, thus generating the final 6HB core structure, typical of the postfusion state (Figure 1D) [1,86,87]. This cascade of refolding steps is believed to be associated with membrane fusion because full 6HB assembly infers that, as mentioned above, the TM domain (embedded in the viral envelope) and the fusion peptide (anchored in the host cell membrane) are brought into immediate proximity, in turn inducing membrane curvature, outer lipid monolayers’ disturbance and ensuing spontaneous membrane merger [88,89,90]. Importantly, the energy released when F refolds from the metastable prefusion state to the thermodynamically highly stable postfusion conformation is thought to overcome the free energy of the lipidic fusion intermediate structure, allowing the creation of the fusion pore.

Interestingly, full completion of the 6HB structure for triggering the paramyxovirus entry process has been recently challenged. Indeed, membrane fusion activity was triggered by MeV F variants displaying two covalently bound HRB domains, thus physically precluding complete 6HB assembly. These data therefore suggested that full completion (and/or proper assembly) of the 6HB structure was not necessarily required for membrane fusion induction and ensuing fusion pore opening [91]. This provides critical information for the design of antiviral drugs with desirable efficacy profiles.

## 6. The F-Activation Stimulus

### 6.1. The Receptor-Binding H Protein: General Information

In contrast to other important infectious pathogens, such as human immunodeficiency virus (HIV), influenza or Ebola virus, most members of the *Paramyxoviridae* family rely on a cofactor for proper activation of the fusion protein: the attachment protein [1]. Therefore, to gain a fundamental knowledge on the cell entry process of these enveloped RNA viruses, it is of critical importance to precisely understand the molecular dynamics employed by the attachment protein to translate receptor binding into F activation.

The attachment proteins of paramyxoviruses evolved in different ways, reflecting their biological adaptation to specific host-receptors. Therefore, for avulaviruses, rubulaviruses and respiroviruses, the attachment protein binds to sialic acid-containing receptors for cell entry [92,93]. These proteins also contain an enzymatic neuraminidase activity that cleaves sialic acid from the progeny virus particles and therefore facilitates the budding event by preventing viral self-agglutination and/or prolonged binding to the host cell. Additionally, these attachment proteins carry the typical hemagglutination activity. For these reasons, such receptor-binding glycoproteins were referred to as hemagglutinin-neuraminidase (HN) proteins. For pneumoviruses, metapneumoviruses and henipaviruses, the attachment protein neither possesses the hemagglutination nor the neuraminidase activities. Thus these attachment proteins have been designated as glycoprotein (G). For morbilliviruses, the attachment protein maintained the hemagglutination function but lost the neuraminidase activity. Such receptor-binding proteins were therefore referred to as hemagglutinin (H) proteins [1].

The attachment proteins of paramyxoviruses are type II integral transmembrane proteins. Hence, each glycoprotein contains a short cytosolic N-terminal cytoplasmic tail and a large extracellular C-terminal domain, connected via a single-spanning hydrophobic TM domain. The extracellular region (or ectodomain) is further divided into a membrane-proximal stalk region and a large membrane-distal cuboidal or globular head domain, presumably linked by a flexible connector.

Upon translation of MeV H monomers in the ER, four units physically associate to form tetrameric structures composed of a “dimer-of-dimers.” Each dimer is built-up by two monomers that are covalently bound via two disulfide bonds located in the upper part of the stalks (Cys-139 and Cys-154) [94]. In contrast to covalent dimers, the dimer-of-dimers interface likely relies on hydrophobic interactions. H oligomerization, glycosylation and assembly with F trimers all occur in the ER compartment and, as stated above, membrane fusion machineries are subsequently exposed on the host cell plasma membrane following the cellular secretory pathway.

### 6.2. Structural Information

#### 6.2.1. H-Heads

Structural studies of several paramyxovirus monomeric H heads (with or without ligands) revealed a common tertiary folding, characterized by a six-bladed beta-propeller conformation [95,96,97,98,99,100,101,102,103,104,105,106,107]. The beta-propeller head contains distinct functional receptor binding sites. For G and HN attachment proteins, the receptor binds to a deep cavity located at the top of the propeller structure [95,98]. Conversely, the MeV H head, despite equally featuring the deep pocket at the top of the barrel, interacts with multiple receptors much more sideways [103,104].

#### 6.2.2. H-Stalks

The only structural information available for any paramyxovirus’ attachment protein stalk domains is derived from hPIV5 and NDV. The atomic structure revealed four α-helices that tetramerize to form a four-helix bundle (4HB). While the upper part of the 4HB folds into a quite straight conformational state (with an 11-mer repeat), the lower part displays a supplementary left-handed supercoiled configuration (with a 7-mer repeat) [105,107]. Importantly, we and other groups have then provided strong evidence that a 4HB-like conformation is the most likely structure also assumed by the morbillivirus H-stalk domain prior to receptor binding [108,109,110].

#### 6.2.3. Tetrameric Attachment Protein Structures

##### MeV H

**Figure 2:**
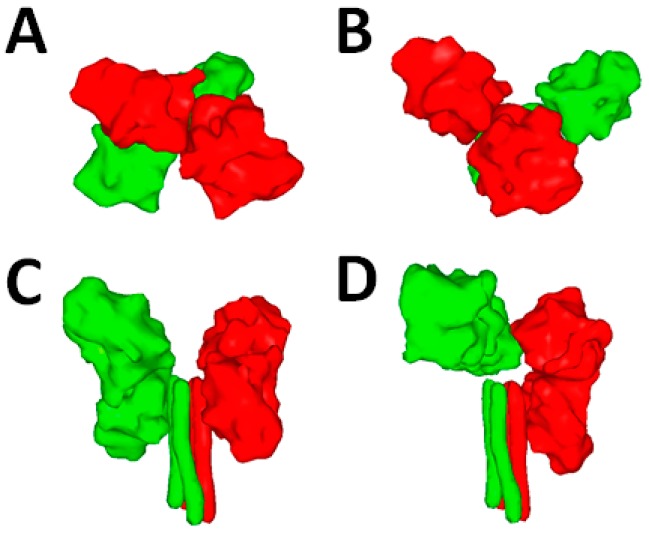
**Possible structure(s) of the MeV attachment protein.** (**A** and **B**) Crystal structures of MeV H in complex with the receptor signaling lymphocyte activation molecule (SLAM) (not shown). Two tetrameric configurations were determined (PDB 3ALZ and 3ALX). High-resolution structures were morphed into low-resolution images using the Sculptor software package; (**C** and **D**) Two alternative folds of H tetramers. Images were obtained from homology models performed with the canine distemper virus CDV) H sequence (strain A75/17) and based on the atomic coordinates of Newcastle disease virus (NDV) HN (PDB 3T1E) and hPIV5 HN (PDB 4JF7). The dimers of the attachment protein tetramer are color-coded in green and red.

Hashiguchi and colleagues determined in 2011 the crystal structure of MeV H-heads in complex with the immune cell SLAM receptor [103]. Furthermore, beyond revealing the precise binding interface, they obtained crystals of MeV H assuming two different tetrameric configurations. In more detail, these atomic structures first revealed that the position of the two monomeric heads forming the dimers was very similar. In contrast, the dimers were assembling differently towards each other in the two tetrameric configurations. Indeed, while in configuration I, the dimers were assembling in a quite planar manner (Figure 2A), in configuration II, they were assembling in a much more staggered way (Figure 2B). This inferred for the first time the notion that the dimers may represent structurally “rigid” units that can “move” as two independent blocks to adopt discrete configurations.

##### NDV HN

The latest structural information of the soluble NDV HN ectodomain was totally unexpected. Not only part of the stalk domain was visible in the crystal for the first time, but the structure additionally illuminated an intriguing positioning of the two dimeric head units. Indeed, while both dimeric heads were not engaged in any tetrameric interface, they were, rather, backfolding onto the stalk with the lower head of each dimer, making a short-range interaction with the upper part of the stalk domain. The latter structure was referred to as the “four-heads-down” conformational state (Figure 2C) [105].

##### hPIV5 HN

The most recent X-ray structure of a soluble form of the hPIV5 HN attachment protein ectodomain featured an intriguing “hybrid” conformational state. In this conformation, only one dimeric unit was backfolding onto the stalk, whereas the other one was positioned in an “up” state due to a helix extending beyond the 4HB-stalk structure. This HN conformation indicated a tetrameric interface between one head of the dimer in the “up” state and one head of the next dimer assuming the “down” state. This HN structure was referred to as the “two-heads-up and two-heads-down” conformation (Figure 2D) [107].

### 6.3. Functional Information

Pioneering work done in the field has demonstrated that membrane fusion activity induced by members of the *Paramyxovirinae* subfamily requires the concerted action of the F and attachment glycoproteins [111,112,113]. While the head domains of the paramyxovirinae attachment glycoproteins were reported to contact host cell surface receptors, compelling functional and biochemical evidence supports the idea that the stalk region of the attachment proteins is the domain predominantly involved in short range interaction with F trimers [1,114,115,116,117,118,119].

Importantly, beyond supporting F-interactions, recent studies reported strong evidence in support of the H-stalk also carrying the F-triggering activity. Strikingly, through consecutive substitution of residues within the stalk domain of CDV and MeV H proteins into cysteines, supplementary disulfide bonds trapping H tetramers could be successfully generated [108,109,110]. Remarkably, when engineered covalent bonds were located within the central region of the H-stalk, bioactivity was blocked and, most importantly, restored when H/F-expressing cells were treated with mild-reducing conditions. These results provided the first functional evidence suggesting that H-heads’ engagement with its cognate receptor triggers structural rearrangements within the central stalk section, which are strictly required for F-activation. Moreover, microdomains within both MeV and CDV H-stalks were mapped: while the F-binding domain (FBD) may consist of residues 110–118, F-triggering may be supported by the broader 85–118 overlapping section [108,114,120,121]. Furthermore, and remarkably, attachment protein constructs lacking the head domains remained, at least partly, functional for most paramyxoviruses (hPIV5, MeV, NDV, MuV, NiV and CDV), thereby clearly spotlighting the critical F-triggering role of the HN, G, and H-stalk domains [122,123,124,125,126].

Additionally, a recent functional analysis of the 167–188 segment of MeV H revealed that this microdomain could accommodate mutations without altering H’s fusion-promotion ability, whereas conversely, insertions or deletions within this segment drastically affected the bioactivity of H-mutants. Combined with the fact that this H-segment remained invariably unresolved in all available MeV H-head crystal structures, the above mentioned functional and biochemical analyses suggest structural flexibility of the 167–188 H-microdomain [127]. Evidently, a flexible linker connecting the stalk to the head domain may offer sufficient structural freedom to enable the proposed receptor-induced repositioning of both H units (head and stalk) that may translate into F activation (see below).

## 7. The Putative F-H Binding Interface

It is well accepted that the paramyxovirus attachment protein is engaged in short-range interaction with the fusion protein. However, no structural data of F in complex with the attachment protein is currently available, thus precluding a comprehensive molecular understanding of the HN/H/G-F mode of interactions. Importantly, the timing of assembly between the attachment and fusion proteins seems to vary considerably among different members of the *Paramyxovirinae* subfamily. For instance, hPIV5 HN may engage with F only at the cell surface, and likely only after HN receptor binding, whereas the morbilliviruses H and F glycoproteins are thought to associate already intracellularly prior to receptor binding [128]. Additionally, henipavirus G and F associate at the cell surface prior to receptor binding [73,129,130], but whether or not they associate intracellularly is still unclear.

**Figure 3:**
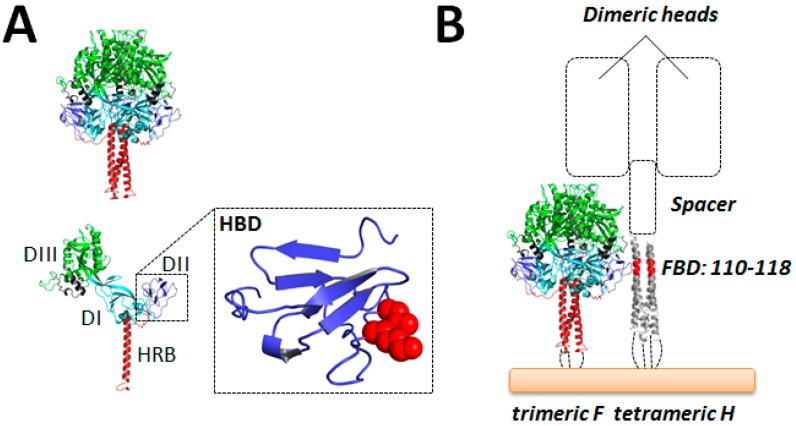
**Model of MeV H/F assembly.** (**A**) Representation of MeV F as a trimer (upper panel) or monomer (lower panel) with the DI, DII and DIII domains color-coded as indicated in Figure 1A. The highlighted box shows a zoom of the Ig-like domain proposed to be important for interaction with H (HBD). The two key hydrophobic residues proposed (in CDV F) to control H interaction are represented as spheres and color-coded in red (l506 and K508) [133]. Images were generated using the PyMol software package (V 1.0, DeLano Scientific, Palo Alto, California, USA); (**B**) Putative H/F assembly. The presumptive HBD of one F-monomer is aligned with the proposed H-stalk segment involved in a short range interaction with F (FBD). The “spacer” H-stalk module, which enables positioning of the two dimeric head units above the F trimer, is represented as a box.

Nevertheless, several functional studies recently highlighted candidate residues in the F heads and attachment protein stalks that may putatively contribute to reciprocal physical interactions. For MeV and hPIV5 F proteins, few hydrophobic amino acids located at the base of the F trimer globular head and near a hydrophobic pocket (formed by two adjacent protomers) were recently identified as candidate H-contacting residues [131,132]. More precisely, these hydrophobic residues were located in a region assuming an immunoglobulin (Ig)-like fold mapping within the DII domain of the F globular head structure (Figure 3A; H-binding domain; HBD). However, while for MeV the strength of the H/F interaction was only partially reduced by substituting these critical residues individually, biochemical evidence (co-immunoprecipitation (co-IP)) could not be provided in the case of hPIV5 F and HN interactions. In a third report, two candidate hydrophobic residues in the CDV F globular head (also located in the Ig-like domain) were demonstrated to also potentially contact CDV H. Once substituted in a combined manner into charged residues, the resulting F-variants exhibited strong impairments in physical association with H (assessed by co-IP), while preserving the prefusion state and substantial cell surface expression profiles [133].

On the other hand, as mentioned above, the H-stalk region 110–118 was identified as a putative candidate region involved in short-range interaction with F (FBD) [114,134]. These findings taken together suggest a staggered model of H/F hetero-oligomeric assembly, where the Ig-like microdomain of the F globular head domain contacts the H-stalk central region (Figure 3B). While this model of H/F assembly infers that the receptor-binding H head modules may be positioned above F trimers, further functional and structural studies are warranted to validate or refute this idea. Supporting this notion, recent evidence suggest that H glycoprotein spikes are “taller” than F glycoprotein spikes, and that this relative height difference is important for the membrane fusion triggering process [134].

## 8. Energetics of F Activation

Concerning the energetics required for F activation mediated by paramyxovirus attachment proteins, the “clamp” and “provocateur” models have been suggested [128,135]. The “clamp” model emerged from the fact that some paramyxovirus fusion and attachment proteins form hetero-oligomer complexes already intracellularly and travel together to the cell surface (as for MeV) [61]. Therefore, it has been hypothesized that, in this case, attachment proteins act as a molecular scaffold that is necessary to maintain F trimers in the metastable, prefusion state, thereby preventing premature activation. This indirectly suggested that such paramyxovirus F trimers would contain low energy barriers in turn precluding proper stabilization of the metastable state in the absence of H. Hence, upon binding of these attachment proteins to a specific host cellular receptor, F would be released from the “clamp”. In turn, F complexes would be destabilized and consequently spontaneously undergo the irreversible refolding cascade.

The “provocateur” model is based on the fact that some paramyxovirus attachment and fusion proteins do not associate intracellularly (as for hPIV5) [136]. Thus, the formation of hetero-oligomeric complexes may occur only as a consequence of attachment proteins binding to a receptor. A conformational modification of the attachment proteins is then required to actively destabilize F trimers. Therefore, the “provocateur” model infers that the prefusion F state of such paramyxoviruses can be maintained alone, thereby relying on a signal from the attachment protein that actively lowers the energy barrier for the initiation of the ensuing spontaneous refolding cascade [135].

Remarkably, taking advantages of conformation-sensitive monoclonal antibodies, the most recent data in the field, however, demonstrated that the morbillivirus and henipavirus F proteins are stable enough to maintain the prefusion state in the absence of H or G [110,137,138]. These findings hence argued against the “clamp” model, while conversely supporting a version of the “provocateur” model for morbilliviruses and henipaviruses. Since H/F and G/F associations are clearly detected before receptor binding, this, in turn, points to some version of the “provocateur” mechanism as a common theme potentially used by all *Paramyxovirinae* to productively trigger F complexes for refolding. Overall, these data taken together clearly infer that morbillivirus H tetramers (and probably most of the *Paramyxovirinae* attachment proteins) must structurally rearrange upon receptor contact to actively trigger F for membrane fusion, even when attachment/F complexes are already previously interacting.

## 9. Latest Models of F Activation

While, as described above, a quite precise mechanistic knowledge on the F refolding cascade to achieve membrane fusion starts to emerge from a panel of mechanistic and structural studies, how H translates receptor binding to F triggering remains largely unknown. However, it is now well accepted that the H protein very likely undergoes some conformational changes as a consequence of receptor engagement, which ultimately lead to F activation. Five main models currently propose how H tetramers may “move” to actively trigger F trimers.

### 9.1. The Sliding Model

**Figure 4:**
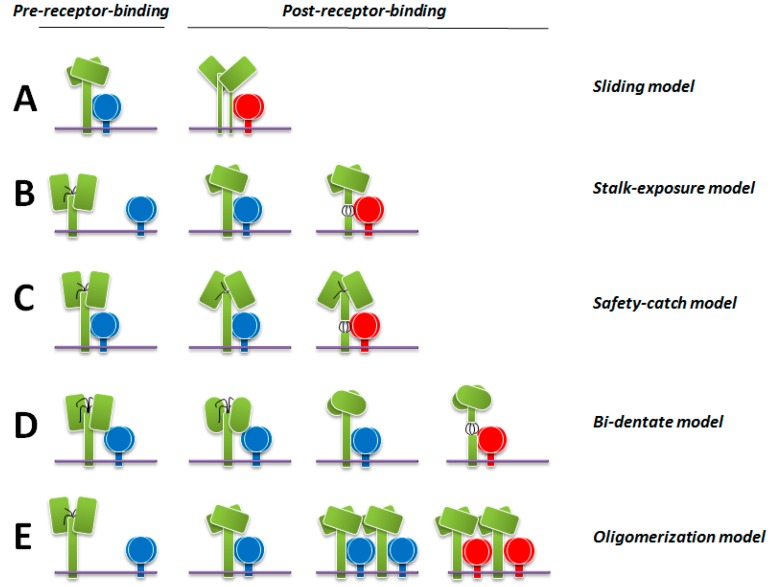
**Models of F activation by paramyxovirus attachment proteins.** (**A**–**E**) Latest models have proposed how attachment protein of different members of the *Paramyxovirinae* subfamily activates F as a consequence of attachment protein-receptor binding. Importantly, all models predict a receptor-induced overall re-positioning of the head regions upon receptor engagements, which may then act as a universal core mechanism for F activation (first described by Bose and colleagues [122]). H tetramers are colored in green. Pre-activated F and activated F are highlighted in blue and red, respectively. In model (**D**) (bi-dentate model) the conformational changes occurring within the head domain prior to heads’ re-positioning are represented by oval structures. The attachment protein stalk regions that are implicated in F activation are shown as black lines (models **B**–**D**).

As mentioned above, the structural data obtained with MeV H illuminated two different tetrameric configurations [103]. Based on these two structures, the authors proposed one model of H-mediated F activation. In this model, H would first fold with the H-heads assuming conformation I (with both dimers associating in a relatively planar configuration) and the stalks adopting the compact “closed” 4HB conformational state. Upon H-heads receptor binding, the H-heads would “slide” laterally to finally achieve conformation II (with dimeric heads assembling in a relatively staggered configuration). As a result, H-stalks would (partially) dissociate and, in turn, lead to the disengagement of F from H. Subsequently, F complexes would be free to undergo the required structural rearrangements to mediate membrane fusion (Figure 4A).

This mechanism infers that MeV H-heads, upon receptor binding, may generate specific signals that would travel through the flexible linkers down to the stalks for efficient F activation. However, latest mechanistic data obtained are not in direct agreement with the sliding model. First of all, head/stalk hybrid attachment proteins were reported to preserve F-triggering activity, provided that the stalks and the F protein originated from the identical virus [139]. Considering that the entire head/receptor framework is swapped in such chimeric attachment proteins, it is difficult to believe that specific receptor-induced conformational changes could be maintained. Secondly, and most importantly, it was recently documented that, for most paramyxoviruses (hPIV5, MeV, NDV, MuV, NiV and CDV), engineered “headless” attachment protein constructs remained bioactive, although to limited extend for some specific constructs [122,123,124,125,126]. We note that fusion promotion by headless attachment proteins was equally induced regardless of the presence or absence of the receptor. These data suggest that the head modules are not necessary to send specific “signals” down to the stalk for productive F activation. However, it is possible that specific signals are sent from the head to the stalk, but that removing the head gets rid of the need for transport of such specific signals. Thirdly, a recent report demonstrated that SLAM-variants displaying mutations in the binding site defined by the interaction of CDV H assuming the putative configuration II, did not substantially modulate H/F-induced membrane fusion activity [140]. Consequently, the biological relevance of the two MeV H-tetrameric conformations for the membrane fusion process deserves future investigations.

### 9.2. The Stalk-Exposure/Induced Fit Model

The second model is based on latest structural and mechanistic information obtained for NDV and hPIV5 HN-proteins. Here, the authors proposed that the “four-heads-down” conformation may represent the initial, pre-receptor-bound, structural state adopted by HN. As described above, this state is characterized by two heads (the lower ones of each dimeric head unit) making physical contacts with the C-terminal region of the HN-stalks and, consequently, directly covering the putative F-binding/activation site [105]. Therefore, such a native conformational state would evidently prevent intracellular HN/F interaction. Upon HN-heads receptor engagement at the cell surface, the dimeric head units would move in an “up” state, which, at the same time, uncover the F-binding/activation site. F would be now free to contact HN-stalks and be activated through a putative induced fit mechanism (Figure 4B) [123,128]. Therefore, this model nicely integrates the surprising data spotlighting the receptor-independent constitutive bioactivity mediated by headless attachment protein constructs: prior to HN-receptor binding, two head modules would inhibit assembly of HN/F complexes, thus preventing premature F activation and ensuing membrane fusion induction. Conversely, in absence of HN-heads, premature glycoproteins’ assembly may occur and induce untimely F activation, thereby eventually promoting membrane fusion in a dysregulated and receptor-independent manner (with F being randomly triggered at the cell surface).

Although the “stalk-exposure/induced fit” model for F activation may present features common to all members of the *Paramyxovirinae* subfamily, a large body of evidence suggest that MeV, CDV, hPIV3 and NiV membrane fusion machineries can already form intracellularly [61,129,141,142]. Additionally, stalk-elongated, and presumably head-lifted, MeV and CDV H constructs remained fully bioactive [126,134] and specific H-stalk mutants exhibit loss of F interactions both in receptor positive and negative cells [108,134], which clearly indicates that some variations of the model may exist.

### 9.3. The Safety-Catch Model

Based on the most recent mechanistic data, it was hypothesized that CDV and MeV H tetramers may initially fold into an auto-repressed conformation, where the H-heads (perhaps by adopting a conformational state resembling the “four-heads-down” HN state) may temporarily lock the inherent F-triggering bioactivity of the stalks by presumptive head-to-spacer stabilizing interactions [62,124,126]. Of note, the morbillivirus H-stalk displays a supplementary segment in the stalk domain (termed “spacer” [109]; Figure 3B), located between the F-binding/activation microdomains and the putative flexible connectors and ensuing cuboidal head modules. This supplementary H-stalk domain may consequently lead to higher positioning of the head domains with respect to the putative F-binding site and thus eventually reducing the probabilities of heads-mediated putative steric hindrances preventing intracellular F-binding. The “safety-catch” model therefore suggests that F complexes can interact with H tetramers without premature activation because H initially assumes an auto-silenced conformation. De-activation of the H auto-repressed state is achieved as a consequence of H-heads’ receptor-binding, in turn leading to re-positioning of the head modules away from the stalks. This results in the disruption of the key stabilizing head-to-spacer interactions, thereby offering freedom to the stalks that will consequently spontaneously undergo structural rearrangements within the central section (also encompassing the F-binding site), which translate into F-triggering (Figure 4C) [62,124,126].

### 9.4. The Bi-Dentate Attachment Protein/F Interaction Model

Liu and colleagues recently introduced another variation of the F-triggering mechanism induced by the attachment protein of NiV [125]. Although the authors identified a receptor-induced “stalk-exposure”-like conformational change in NiV G, both glycoproteins were previously reported to assemble already intracellularly, as in the case of MeV and CDV membrane fusion machineries. Here, a “bi-dentate” attachment protein/F interaction mechanism was hypothesized to exclude premature F activation upon early G/F interaction. This model predicts that, initially, NiV F may bind to the attachment protein head domains. NiV G engagement to its cognate receptor would then trigger conformational changes in G (including two occurring in the G head domain and followed by a “stalk-exposure”-like movement) that enables F to switch, or relatively gain, interactions from the heads to the stalks, in turn resulting in F activation (Figure 4D) [125]. Noteworthy, this model is not mutually exclusive with the “safety-catch” model, which could also explain the current NiV data.

Although possible for NiV F activation, recent data obtained for CDV and MeV H proteins are not in agreement with this hypothesis. As already mentioned above, H tetramers with elongated stalks remained bioactive and H-stalk variants lost binding competence to proteolytically matured F trimers, even in receptor-negative cells [62,108,134]. Moreover, “headless” H constructs retained intracellular F interaction [62]. Whether NiV F is activated by G through the proposed “bi-dentate” mechanism or via a mechanism more closely resembling the one hypothesized for morbilliviruses (“safety-catch” model) remains to be determined.

### 9.5. The Receptor-Induced Oligomerization Model

Recent EM observations of hPIV3 HN/F complexes anchored on virions’ envelopes suggested that both glycoproteins may associate already prior to receptor engagement. Additionally, a sub-population of HN-proteins, in complex with F, were displaying “heads-up”-like configurations, in turn inferring that the HN “heads-up” conformation is not sufficient to activate F [143].

Using bimolecular fluorescence complementation technology, Porotto *et al.*, additionally obtained a series of data suggesting clustering of preformed HN/F complexes upon receptor engagement [144]. Taken together, these data led the authors to propose an alternative model whereby HN may transmit a signal for F activation that is triggered by a receptor-induced H-oligomerization mechanism (Figure 4E) [143]. However, although a similar mechanism was proposed for hPIV5 years ago [145,146,147], this model currently lacks experimental evidence confirming that monomeric, dimeric or tetrameric HN-structures can switch to higher order oligomers as a result of interaction with the receptor to ultimately achieve conformations productive for F activation.

Taking together, it becomes increasingly evident that, to gain further key information of the membrane fusion process, acquisition of novel structural information is mandatory. More specifically, the determination of any paramyxovirus attachment protein in complex with F at atomic, or near-atomic, resolution would certainly highlight instrumental insights of the molecular mechanism sustaining paramyxovirus cell entry. Of note, high-resolution structural information can be now gathered using cryo-transmission electron microscopy (TEM) and single particle 3D reconstruction from electron micrographs recorded with the revolutionary direct electron device (DED) technology. This may set an innovative framework not only to obtain structural information of HN/H/G-F complexes to unprecedented levels, but also to deliver critical information for antiviral drug design.

## 10. The Concept of the F-Triggering Range

Recently, we and others have proposed that the capacity of lowering the intrinsic energy barrier of the prefusion F state varies depending on the particular triggering stimulus [148,149]. Such stimulus relies on the molecular nature of H and the origin of the cellular receptor. The combination of the triggering stimulus with the intrinsic F protein metastability then defines the so-called “F-triggering range”, or the capacity of a given F to be activated to mediate membrane fusion in a given cellular environment. In other words, we predict that the specific contacts between H tetramers and a host cell receptor open a range of F-triggering possibilities. If the F protein contains an inherent energy fitting within this range, then H can lower the energy barrier of such F complexes to levels sufficient for their destabilization and activation [148].

Therefore, we propose that the H/SLAM interaction leads to the opening of a wide F-triggering range, thus enabling even highly stable F complexes to be destabilized for membrane fusion. On the other hand, the H/N4 association results in a narrower F-triggering range. Thus, in such environmental conditions, highly stable F complexes would not be activated at all or only to levels displaying only limited cell-cell fusion efficiency [148]. As a matter of fact, for a given virus, massive syncytia formation is observed in Vero-SLAM cells compared to those formed in Vero-N4 cells (when very similar amounts of receptors are expressed at the cell surface), an *in vitro* situation that seems to correlate with *in vivo* observations.

The A75/17-CDV strain is highly neurovirulent: it can induce demyelinating encephalitis associated with persistent infection of astrocytes [150,151,152]. As mentioned above, we obtained evidence that an unknown molecule may act as a functional receptor for CDV in astrocytes (referred to as GliaR) allowing cell entry and spread [59]. Therefore, we hypothesize that the H/GliaR mode of interaction provides a suboptimal F-triggering range. The latter combined with the exceptionally high intrinsic stability of A75/17-F will translate into poor membrane fusion triggering presumably restricting cell-cell fusion to microfusion pores and, in turn, allowing swift transfer of ribonucleocapsids between cells. This mechanism may eventually explain how CDV “outruns” the immune system and leads to viral persistence associated with a unique pattern of progressive multifocal white matter destruction.

## 11. Development of MeV F Inhibitors

Because MeV-mediated clinical symptoms are, at least in part, immunopathological in nature and occur at a time when viral clearance is already advanced, the usage of antivirals to cure full blown measles is probably unproductive. Nevertheless, it was recently proposed that MeV antiviral drugs may potentially synergize with current vaccination programs to achieve the threshold of 95% herd immunity that is required for successful global MeV eradication. Hence, post-exposure prophylaxis of people exposed to confirmed cases of measles may potentially help to contain epidemics [3].

Importantly, although neurological complications of MeV infection such as SSPE and MIBE are rare, they often remain unrecognized in developing countries because these complications occur months to years after the acute infection [35]. However, since SSPE and MIBE are crippling and lethal diseases, the potential effectiveness of inhibitors in patients with confirmed MeV-induced neurological complications should be investigated. We note that two promising candidate inhibitors were recently proven to protect against virulent morbillivirus challenge in established animal models: a small-molecule anti-polymerase inhibitor and a peptidic entry inhibitor [23,24,25]. This underscores the need for the development of new inhibitory molecules, which would be of utmost importance for combined antiviral therapeutic strategies to prevent the rapid emergence of drug-resistant viral variants.

### 11.1. Anti-F Peptidic Inhibitors

As mentioned above, the MeV F protein, like all class I viral fusion proteins, contains two conserved heptad repeat (HRA and HRB) regions. While HRA locates near the *N*-terminal region of the proteolytically mature form of the membrane-anchored F1 subunit, HRB is found proximal to the C-terminal TM domain of the identical subunit. As first noted for the HIV gp41 glycoprotein [153,154], peptides derived from either heptad repeat have been shown to act as potent inhibitors of the fusion process [153,155,156,157,158,159,160]. It is widely accepted that HRB-derived peptides target a structural intermediate (presumably the HRA-derived extended coiled-coil of pre-hairpin F) that transiently emerges during the refolding cascade, thereby interfering with formation of the 6HB-carrying central core of the final postfusion state.

As first demonstrated for HIV, NiV, and hPIV3, increased inhibitory efficacy was obtained by conjugating a cholesterol group to the peptides [161,162,163]. Such modified peptides could efficiently prevent lethal challenge with NiV in mice [161]. Importantly, similarly conjugated MeV F HRB-derived peptides were recently documented to also potently inhibit viral spread in brain explants as well as in an animal model recapitulating MeV encephalitis [25]. Based on the latter modifications, the anti-F peptidic entry inhibitor was demonstrated to (i) exhibit longer half-lives; (ii) display high efficacy after simultaneous subcutaneous and intranasal administration; and (iii) efficiently cross the blood-brain-barrier (BBB), indicating a possible therapy for MIBE and SSPE. Assuming that viral transmission in the brain would indeed rely on functional F proteins completing a *bona fide* refolding process to induce membrane fusion for transmitting the ribonucleocapsid from one cell to another, anti-F peptidic inhibitors could potentially abrogate this process in patients with MeV-induced neurological diseases.

### 11.2. Anti-F Inhibitory Antibodies

Several highly efficient neutralizing monoclonal antibodies (nAbs) targeting MeV F-trimers were produced decades ago [164,165]. Importantly, while passive immunization (with immunoglobulins) within seven days of exposure may prevent measles [166], treatment with specific anti-F nAbs were not only neutralizing but able to passively protect mice [164]. Interestingly, it has been only recently reported that some of the generated anti-F nAbs were specifically recognizing the prefusion F conformational state [137]. Although the molecular mechanism of neutralization remains undetermined, it is conceivable that the nAbs either over-stabilize prefusion F and/or physically restrict the initiation of the large structural rearrangements. Alternatively, they may also lead to untimely H/F dissociation. Of note, not all prefusion F-recognizing mAbs are neutralizing. Indeed, a prefusion CDV F-specific monoclonal antibody could neither inhibit viral cell entry nor cell-to-cell spread [137]. This underscores the intriguing concept that some epitopes within the metastable state of F trimers can accommodate binding of one (or more) monoclonal antibody with little to no impact on the series of irreversible large-scale conformational changes that lead to membrane fusion.

Independently of the mechanism of inhibition, considering that infants and young children are the primary target of anti-measles therapy, monoclonal antibodies may not exhibit a suitable pharmacological profile. First, as mentioned above, immunopathological reactions in fulminant MeV infections will not respond to, or even be aggravated by, this treatment. Second, if used for post-exposure prophylactic therapy, repetitive injections of the monoclonal antibody would require well-functioning healthcare systems. Third, since monoclonal antibodies hardly cross the BBB, their effectiveness to treat neurological complications would be certainly quite limited. Finally, an ideal drug profile should also meet a reasonable cost/efficiency ratio, which might be challenging to achieve with monoclonal antibody-based therapies, as for instance experienced with the high cost of the anti-RSV-F palivizumab antibody [167,168].

### 11.3. Anti-F Small-Molecule Blockers

Recently, Plemper and colleagues successfully developed a unique class of MeV fusion protein small-molecule blocker (AS-48) [169]. The compounds were rationally designed based on a modelled structure generated from the atomic coordinates of NDV F postfusion state (no prefusion state of any paramyxovirus F proteins was available at this time). Importantly, although the F protein used as a template was derived from the Edmonston vaccine strain, it was subsequently demonstrated that the AS class of antiviral drugs exhibited broad-spectrum efficacy; not only the compounds could block membrane fusion induced by multiple MeV strains (including wild-type isolates), but cell entry and spread mediated by various CDV strains were similarly impaired, thus highlighting the potent *pan*-morbillivirus inhibitory ability of this class of entry inhibitors [170,171,172].

Because a wide pocket located within the stalk domain of the postfusion F structure was originally employed as a guide for drug design, it was assumed that the antiviral compound could bind to a target site in a transiently emerging late F structural intermediate, in turn interfering with the transition to the final postfusion state. Surprisingly, additional data were rather consistent with the AS-48 class of morbillivirus fusion inhibitors exerting a stabilizing activity on prefusion F trimers (see below).

In any case, it is important to note that, despite (i) putative interaction with the prefusion F state (substantially increasing the window of opportunity for compound interference); (ii) excellent efficacy profiles even against CDV-mediated non-cytolytic spread in highly sensitive primary canine astrocytic cultures [172]; and (iii) *pan*-morbillivirus efficacy [170,171,172], only intermediate antiviral potency could be achieved in cultured cells (with low μM range efficacy) [172,173], which compromised further profiling of the AS-48 small-molecule compound and derivative analogs in animal models.

### 11.4. Mechanism of Viral Resistance to AS-48 and Chemical Analogs

A complete understanding of resistance mechanisms must be acquired for clear decision-making before the use of any novel antiviral candidates. As a matter of fact, the high inherent mutational rate of RNA virus polymerases [174,175] favors the swift emergence of drug-resistant viral variants.

Regarding the AS-48 fusion inhibitor class, drug-resistant viral variants were efficiently generated in cultured cells. Viral resistance to AS-48 can be achieved, at least in part, through mutations at MeV F positions 462 and 367 [176]. In a postfusion F structural homology model, residue N462 (mapping to the C-terminal region of HRB) contributes to the overall assembly of the 6HB structure, whereas residue A367 (mapping within the DI domain) is located at the tip of the head domain, roughly 100 Å away from residue N462. In sharp contrast, in a prefusion F model, both residues are in close proximity (alpha carbons with less than 13Å distance) and localize at the structural transition of the short stalk and the large globular head domains [177].

Concerning the mode of action of the fusion inhibitor, initial findings supported the hypothesis that AS-48 docks into a pocket microdomain that transiently emerges in a late F structural intermediate, thus preventing membrane fusion by interfering with proper transition to the postfusion conformation. Drug design based on a postfusion F model and escape mutations mapping to the HRB region known to contribute to 6HB formation favored a primary site resistance mechanism, which would compromise high affinity interaction of the compound with the target site. However, although substitutions in HRB emerged, neither naturally-occurring mutations in the opposite HRA binding partner were identified, nor rationale substitutions at these HRA positions did result in drug-resistant F-mutants, which rather supported the hypothesis of a secondary resistance mechanism [176,177].

Further complicating the interpretation of the data, resistance to AS-48 also correlated with the emergence of mutations leading to F complexes characterized by a destabilizing phenotype [176]. Interestingly, while mildly destabilized F-variants turned hyperfusogenic, the most severely destabilized F complexes exhibited both improper cell surface transport competence and fusion-deficiency. Remarkably, for those latter mutants, cell surface expression and ensuing bioactivity could be reversed in the presence of the drug [176]. This prompted Prussia and colleagues to propose a mechanism of viral resistance relying on accelerated F-refolding kinetics, which would translate in narrowing the window of opportunity for compound interference with a transiently emerging target site [177].

Strikingly, a similar mechanism of resistance was recently proposed for RSV to efficiently escape a panel of structurally diverse small-molecule fusion protein inhibitors, thus potentially spotlighting a *pan*-paramyxovirus resistance mechanism [178]. On the other hand, Battles *et al.*, very recently determined the structure of RSV prefusion F in presence or absence of various antiviral drugs (JNJ-2408068, JNJ-49153390, TMC-353121, BTA-9881, and BMS-433771 [179,180,181]). Remarkably, despite displaying structurally diverse scaffolds, all compounds docked into a three-fold-axis central pocket locating within the internal side of the prefusion F structure. They provided structural evidence that the inhibitors-escape mutations either mapped to amino acids directly interacting with the compounds or to the immediate neighboring ones that require conformational change to enable the compounds to dock into the pocket [182]. Importantly, although drugs-escape mutations occurred in similar F microdomains in both works, most of the reconstituted F-mutants in the latter study did not exhibit hyperfusogenic phenotypes [182]. These data thus underscored three possible mechanisms of resistance: two directly involved in preventing compounds high-affinity interaction (primary site resistance model) and one relying on the overall destabilization of the metastable F conformation (kinetics resistance model).

However, although possible for MeV F, the kinetics resistance model hardly explains why the compound could nevertheless potently restore surface expression and bioactivity of inherently destabilized F-mutants [176]. In fact, these findings additionally strongly supported the hypothesis that the compound could still bind efficiently to F, which consequently also argued against a primary site resistance mechanism. Three key additional molecular insights on the mode of action exerted by AS-48 and the 3g chemical analog were recently provided. First, using newly identified conformation-sensitive monoclonal antibodies, Ader and colleagues clearly demonstrated that 3g efficiently stabilized the prefusion state of inherently destabilized F-mutants [137]. Second, refolding of wild-type F trimers occurred at a substantially higher temperature in the presence than in the absence of the drug [137]. Third, 3g prevented fusion induction presumably without blocking F-refolding *per se*, since H/F-mediated fusion inhibition by 3g could be overcome by elevating the amount of environmental energy [137].

Taken together, several lines of evidence support the notion that AS-48 and chemical analogs preferentially bind to the prefusion-like F conformation rather than to a target site transiently emerging in late structural intermediates [137,176]. In turn, the antiviral drug will exert a strong stabilizing impact on metastable complexes, which translates in preventing productive H-mediated F-triggering activity. Viruses with over-stabilized prefusion F trimers tend to accumulate mutations in specific F microdomains resulting in increasing the inherent energy of the metastable state up to levels re-accommodating H-mediated F-triggering. We speculate that the putatively faster refolding kinetics and ensuing hyperfusogenicity associated with some F mutants in the absence of the drug may represent the consequence rather than the cause of the resistance mechanism. In fact, we hypothesize that the ensuing cascade of conformational changes (triggered if metastable F trimers display the appropriate inherent energy) may be sterically and/or thermodynamically impaired by the compounds occupying its target site, thereby potentially counteracting the putative mutations-driven faster refolding kinetics.

Remarkably, the prefusion state-destabilization resistance model infers that some escape F-mutants may even turn to be dependent on the antiviral compound to induce membrane fusion. In fact, this is exactly the phenotype observed with the most severely destabilized F-variants: while in the presence of the drug bioactivity was restored, in the absence, the prefusion state of such F trimers was so labile that they spontaneously refolded into the postfusion state, thereby expressing only bioactive-dead trimeric complexes at the cell surface [137,176].

Overall, regardless of the precise viral resistance mechanism against AS-48 class of entry inhibitors, a large body of mechanistic and structural evidences illuminates “F trimer-destabilization” as one possible common theme used by paramyxovirus fusion proteins to escape structurally diverse viral entry blockers [137,176,178]. Although any residues mapping along the broad protomer-protomer interfaces may potentially destabilize trimeric F proteins, recent data strongly suggested the presence of specific microdomains critical for proper stability of the prefusion state [137,148,176]. Corroborating these data, a recent report by Poor and colleagues revealed, by oxidative footprinting assays, the presence of critical microdomains involved in stabilizing the prefusion state of PIV5 F and ensuing putative conformational changes required to achieve the postfusion conformation [183]. The existence of hot spot prefusion conformation-stabilizing microdomains, which can accommodate mutations to deliver *pan*-antiviral resistance, is strongly questioning the efficacy of targeting paramyxoviral fusion proteins for the development of novel therapeutic options.

Although further research is necessary to identify definitive therapeutic drugs for use in MeV infections, there are several promising targets and approaches. Importantly, pathogenicity of escape viral variants must be carefully investigated in animal models to ensure that the emerging mutations do not translate in enhanced virulence profiles, in particular with viruses developing hyperfusogenic F proteins. In fact, although human clinical investigations were not performed, RSV variants which developed *pan*-resistance to antiviral drug treatment remained fully pathogenic in a mouse model [178]; a phenotype that may nevertheless be strain-specific [182].

## 12. Functional Impact of the F Protein in Brain Disorders: A Highly Regulated or Dysregulated Membrane Fusion Process?

Morbilliviruses are well known to be neurovirulent pathogens [34,35]. More than 50% of patients with measles display abnormal electroencephalograms [184,185]. However the appearance of clinical neurological disease remains rare in MeV infection. Nevertheless, as a result of viral persistence in the brain, MeV can induce neurological diseases, such as SSPE or MIBE. While the former can occur years after the acute phase of the disease in an otherwise normal patient, the latter may develop several months following initial exposure in immunocompromised patients [186,187].

A panel of studies revealed that brain-isolated SSPE viruses (SSPE strains) accumulated extensive mutations, which are thought to be potentially associated with neuro-virulence. Of particular importance, mutations and/or deletions affecting the structural M, F and H proteins (well-known components controlling viral assembly and budding) are often detected in SSPE strains [188,189,190,191,192]. Consequently, such viral variants are defective in producing infectious progeny and it is generally assumed that ribonucleocapsids are transmitted from an infected cell to a naïve neighboring cell by way of microfusion pores [55,56,57] (also referred to as intercellular membrane pores [58]).

While alterations in the cytoplasmic tail of F are considered a critical factor leading to budding-deficient viruses and ensuing neurological disorders, recent reports identified several substitutions in the F-ectodomain, which potentially may also confer neurovirulence. Indeed, recombinant wild-type MeV carrying the mutations F-T461I or F-S103I/N462S/N465S exhibited neurovirulence in suckling hamsters [193,194]. Importantly, functional and biochemical analyses of the F-variants revealed that the mutations destabilized the metastable, prefusion conformation, which consequently conferred hyperfusogenic phenotypes in SLAM- and N4-expressing cells. Remarkably, such F complexes even gained the ability to induce syncytia formation in cells lacking the two main morbillivirus receptors [194]. Furthermore, by introducing the mutation L454W in MeV F (a mutation identified in two HIV-infected individuals additionally diagnosed with MIBE) [195], Jürgens and colleagues recently confirmed the intriguing notion that F-variants of brain-derived MeV strains are associated with destabilized and hyperfusogenic phenotypes [196]. This is of crucial importance with regard to the future development of (and/or investigation of available) F inhibitors to eventually treat MeV CNS infections, since brain-derived MeV viruses with destabilized F-variants may exhibit the *pan*-resistance mechanism described above.

In sharp contrast, efficient cell-to-cell spread of CDV in the CNS of dogs occurs with wild-type fusion complexes. Indeed, no mutations specifically associated with CNS-isolated CDV strains are known. Additionally, inoculation experiments with recombinant wild-type CDV in primary canine brain cell cultures demonstrated that the virus could efficiently spread from cell-to-cell in a non-cytolytic manner without the requirement of any mutations in the entry machinery [52,53]. Rather, the findings supported the hypothesis that the CDV entry complex remained functional [59]. However, in the CNS, the CDV fusion machinery is likely activated in a very selective manner, perhaps as a consequence of (i) suboptimal interaction of H with a putative receptor expressed in astrocytes (GliaR) and/or (ii) the recent discovery that wild-type prefusion F complexes are very stable [148]. Hence, for CDV, microfusion pore formation would be the result of highly stable prefusion F complexes being poorly activated by the low stimulus generated by H/GliaR contacts. Conversely, perhaps as a result of the lack of candidate receptors for MeV in neurons, efficient lateral cell-to-cell spread may rely on the accumulation of mutations in F trimers, causing the destabilization of the prefusion conformation down to levels that bypass the direct requirement of signals promoted by the H/receptor interactions.

Taken together, although persistence in the brain seems to be a mechanism common to both MeV and CDV to trigger neurological diseases, recent studies with MeV argue for the requirement of a dysregulated fusion machinery to support the establishment of persistent CNS infections. In contrast, recent data obtained with CDV argue for the hypothesis that highly regulated fusion machineries are necessary to enable non-cytolytic cell-to-cell spread. It is thus tempting to speculate that these contrasting findings may, at least in part, contribute to the well-known large difference in frequency of neurological complications triggered by MeV and CDV infections in their respective hosts.

## 13. Conclusions and Perspectives

Research in recent years leaves no doubt that membrane fusion induced by morbilliviruses is not only the first critical step of the infection but also determines cell pathology in specific tissues and hence the course and outcome of the disease. The molecular and functional understanding of morbillivirus-associated membrane fusion has progressed to the point where it has become feasible to control this process by treating the fusion protein with inhibitory molecules. The variety of approaches aiming to develop anti-F drugs combined with the gained knowledge on derived drug resistance mechanisms [137,148,176,177], however, strongly suggest that combined therapies will be a prerequisite for the development of entry inhibitors. Therefore, discovery of additional anti-F small-molecule compounds with different inhibitory mechanisms and/or identification of anti-H inhibitors may translate into a realistic therapeutic option in the near future. The primary aim will be post-exposure treatment to contain local outbreaks by complementing WHO vaccination campaigns that aim to eradicate measles [3]. A further potential application is the treatment of severe, particularly neurological, MeV infections, which, albeit rare, are invariably crippling and lethal diseases.
